# Local ecological divergence of two closely related stag beetles based on genetic, morphological, and environmental analyses

**DOI:** 10.1002/ece3.8837

**Published:** 2022-04-17

**Authors:** Sheng‐Nan Zhang, Kôhei Kubota

**Affiliations:** ^1^ Department of Forest Science Graduate School of Agricultural and Life Sciences The University of Tokyo Tokyo Japan

**Keywords:** character displacement, environmental niche, intraspecific variation, mitochondrial introgression, *Platycerus delicatulus*, *Platycerus kawadai*

## Abstract

The process of phenotypic adaptation to the environments is widely recognized. However, comprehensive studies integrating phylogenetic, phenotypic, and ecological approaches to assess this process are scarce. Our study aims to assess whether local adaptation may explain intraspecific differentiation by quantifying multidimensional differences among populations in closely related lucanid species, *Platycerus delicatulus* and *Platycerus kawadai*, which are endemic saproxylic beetles in Japan. First, we determined intraspecific analysis units based on nuclear and mitochondrial gene analyses of *Platycerus delicatulus* and *Platycerus kawadai* under sympatric and allopatric conditions. Then, we compared differences in morphology and environmental niche between populations (analysis units) within species. We examined the relationship between morphology and environmental niche via geographic distance. *P*. *kawadai* was subdivided into the “No introgression” and “Introgression” populations based on mitochondrial *COI* gene – nuclear ITS region discordance. *P*. *delicatulus* was subdivided into “Allopatric” and “Sympatric” populations. Body length differed significantly among the populations of each species. For *P*. *delicatulus*, character displacement was suggested. For *P*. *kawadai*, the morphological difference was likely caused by geographic distance or genetic divergence rather than environmental differences. The finding showed that the observed mitochondrial–nuclear discordance is likely due to historical mitochondrial introgression following a range of expansion. Our results show that morphological variation among populations of *P*. *delicatulus* and *P*. *kawadai* reflects an ecological adaptation process based on interspecific interactions, geographic distance, or genetic divergence. Our results will deepen understanding of ecological specialization processes across the distribution and adaptation of species in natural systems.

## Graphical abstract

We examined the evolutionary history of two closely related stag beetle species *Platycerus delicatulus* and *P*. *kawadai* across their known distribution ranges in Japan. We evaluated morphological and environmental differentiation to reveal ecological specialization resulting from local adaptation. Our results suggest that interactions between interspecific relationship and dispersal may promote niche divergence of closely related beetles.

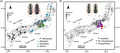

## 1 INTRODUCTION

How and why the diversity of life on earth increased over time are key research questions in ecology and biogeography (Blanquart et al., 2013; Cox et al., 2016; Futuyma & Antonovics, 1992; Savolainen et al., 2013; Thomas et al., 2016). Genetic and ecological speciation can occur in different parts of an ancestral species’ range in which contrasting environmental conditions lead directly or indirectly to the evolution of reproductive isolation (Faulkes et al., 2004; Rundle & Nosil, 2005; Schluter, 2001). However, genetic divergence within and among species does not always cause divergence of morphological and other phenotypic traits due to silent mutations and phenotypic convergence (Fitch, 1970; Ujvari et al., 2015). Adaptative phenotypic variation often occurs via the evolution of eco‐morphological innovations that contribute to ecological specialization in response to environmental variations or interspecific interactions (Devictor et al., 2010; Mammola et al., 2020). Therefore, evaluation of the phylogenetic constraints on traits and trait–environment relationships can elucidate the mechanisms underlying evolutionary selection and their impact on current ecological patterns.

Phenotypic adaptation among environments is recognized in a wide variety of taxonomic groups (Benito Garzón et al., 2011; Ghalambor et al., 2007; Pavlek & Mammola, 2021; Xue et al., 2019). Considering adaptation via multivariate genetic and trait analyses is essential in such situations. However, comprehensive studies integrating phylogenetic, phenotypic, and ecological approaches to assessing speciation process and identifying phenotypic variations correlated with local adaptation have usually been neglected.

**FIGURE 1:**
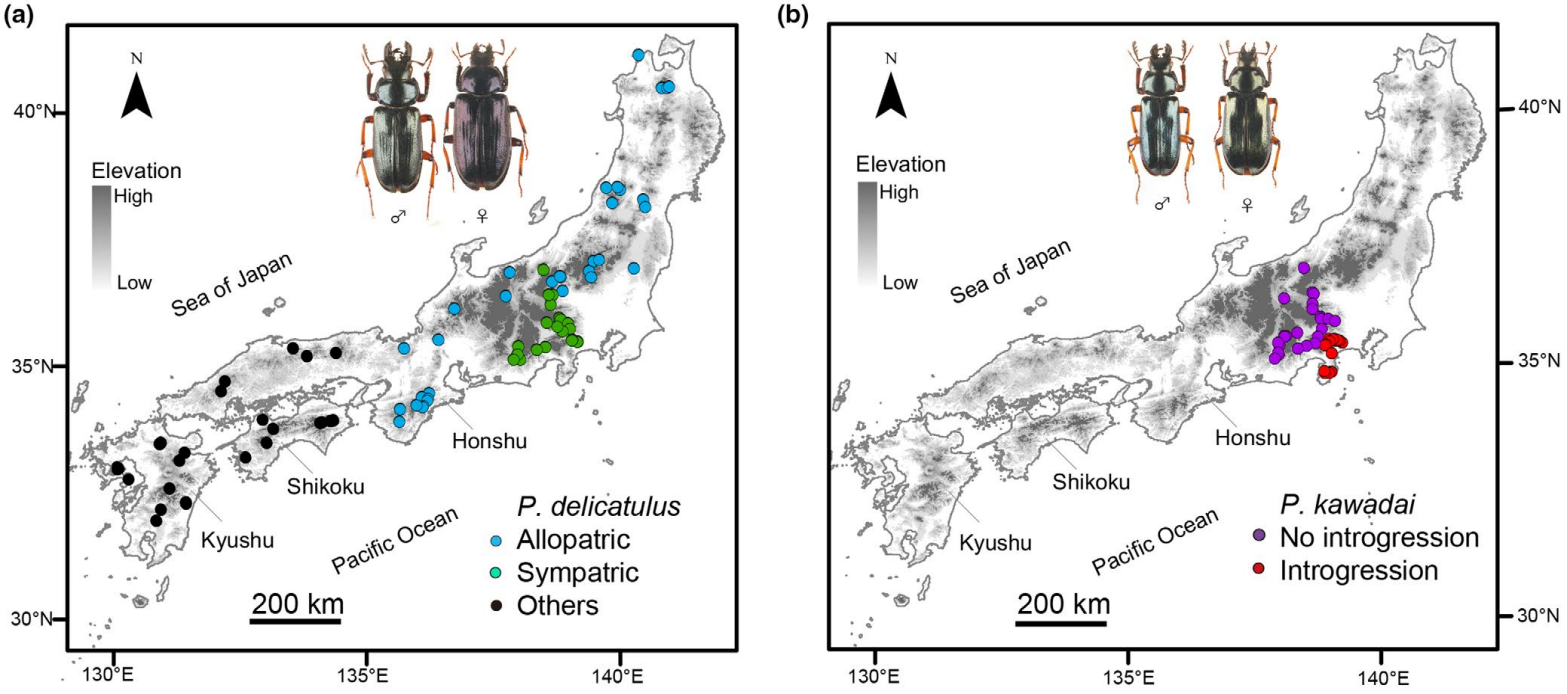
Occurrence records of *Platycerus delicatulus* (a) and *Platycerus kawadai* (b) at the collection sites in Japan

Here, we investigated inter‐ and intraspecific relationships using genetic, morphological, and ecological data for two closely related *Platycerus* beetles, *Platycerus delicatulus* Lewis, 1883, and *Platycerus kawadai* Fujita and Ichikawa, 1982, to explore how local adaptation shapes their habitat preference. *P*. *delicatulus* and *P*. *kawadai* of the family Lucanidae are endemic to Japan and exhibit geographic genetic variations (Kubota et al., 2011). Both species prefer mature cool temperate deciduous broad‐leaved forests. *P*. *delicatulus* has a wide distribution across the main islands of Japan, except Hokkaido. *P*. *kawadai* appears to be endemic to central Japan (Figure 1). Both species co‐occur throughout the range of *P*. *kawadai*, although some differences in host wood preference have been observed. More specifically, *P*. *delicatulus* and *P*. *kawadai* prefer hard and dry decaying wood as their larval resources, whereas all other *Platycerus* species in Japan prefer soft and wet decaying wood on the forest floor. However, *P*. *delicatulus* is more abundant at lower elevations, especially on thick decaying wood, and *P*. *kawadai* tends to target thin decaying wood at higher elevations (Kubota et al., 2020). Two species would lose large portions of present suitable area under climate change (Zhang & Kubota, 2021). Phylogenetically, the two species diverged approximately 1 million years ago, and no hybridization between them has been recorded (Kubota et al., 2011; Zhu et al., 2020). *P*. *delicatulus* and *P*. *kawadai* are sister species with similar morphological and ecological attributes, such that sympatric distributions might lead to ecological divergence. Congeneric and ecologically similar species are considered good models for studies of ecological divergence, and thus these two species provide an opportunity to explore mechanisms underlying niche evolution and develop policies for insect management and conservation strategies.

The present study aimed to quantify multidimensional differences among populations that may arise due to local adaptation in the closely related species *P*. *delicatulus* and *P*. *kawadai*. First, we estimated the intra‐ and interspecific evolutionary dynamics of these two species across their ranges and constructed intraspecific analysis units using integrated phylogenetic results for both species under sympatric or allopatric conditions. We then explored differences in morphology and environmental niche among the populations within each species. We examined the relationship between morphology and environmental niche via geographic distance to assess whether local adaptation may explain population differentiation.

## 2 METHODS

### 2.1 Molecular procedures and phylogenetic analyses

This study focused on *P*. *delicatulus* and *P*. *kawadai* individuals collected from 2005 to 2020 for genetic analysis across the entire geographic range of these two species (Figure 1). The collection sites of the two species are listed in Appendix 1. Besides, *Platycerus akitaorum* Imura, 2007, and *Platycerus sugitai* Okuda & Fujita, 1987, were used as outgroups.

In this study, we determined 94 and 45 sequences of the mitochondrial cytochrome oxidase subunit I (*COI*) gene and nuclear internal transcribed spacer (ITS) region, respectively (Appendix 2). Genomic DNA was extracted from the testis or muscle tissues of adult beetles or larvae preserved in absolute ethyl alcohol using the Wizard Genomic DNA Purification kit (Promega).

We amplified fragments of the *COI* gene (primers C1‐J‐2183 and L2‐N‐3014, Simon et al., 1994) and ITS region (primers 5.8S38F and ITS4col, Tanahashi & Hawes, 2016) to explore the phylogenetic relationships within and between the two species. *COI* was amplified by polymerase chain reaction (PCR) at 94°C for 3 min, followed by 30 cycles of 94°C for 1 min, 48°C for 1 min, and 72°C for 1 min, and a final extension for 7 min at 72°C. The ITS region was amplified using the same process, but with an annealing temperature of 50°C. The PCR products were purified using the Illustra ExoStar Clean‐Up kit (GE Healthcare).

Additionally, we used 65 *COI* and 5 ITS sequences for *P*. *delicatulus* and *P*. *kawadai*, and 9 *COI* and 2 ITS sequences for the outgroup (*P*. *akitaorum* and *P*. *sugitai*) from previous studies (Kubota et al., 2010, 2011; Zhu et al., 2020). In total, we used 168 *COI* and 52 ITS sequences for analysis. The best‐fit substitution model for *COI* and the ITS region were selected using jModelTest v.2.1.10 (Darriba et al., 2012) based on the Akaike information criterion (AIC).

Bayesian interference (BI) trees were constructed using MrBayes v.3.2.7 (Ronquist et al., 2012) for 100 million generations (sample frequency = 50,000) with Tracer v.1.7.1 (Rambaut et al., 2018). After discarding the first 10% of samples as burn‐in, majority‐rule consensus (MRC), trees were constructed by the sumt function in MrBayes. The final tree was visualized using FigTree v.1.4.2 (Rambaut, 2016). Maximum‐likelihood (ML) trees were constructed using RAxML v.8.2.9 (Stamatakis, 2016) with the best‐fit substitution model selected using 1000 bootstrap replications.

Divergence times were estimated using BEAST v.2.6.2 based on the strict molecular clock with a substitution rate of 1.77% per lineage in million years (Myr) for *COI* (Papadopoulou et al., 2010). The data consisted of only in‐group taxa, and the topology was fixed to the ML tree. Markov Chain Monte Carlo analysis was performed using 10 million generations, sampling every 1000 generations. The convergence of the chains was confirmed using Tracer v.1.7.1. After discarding 10% of samples as burn‐in, samples from the posterior distributions were summarized on a maximum clade credibility tree using TreeAnnotator v.1.10.5. FigTree v.1.4.2 was used to visualize the resulting tree.

Based on the molecular analysis results, we subdivided the populations of *P*. *kawadai* into two analysis units (see RESULTS). For *P*. *delicatulus*, we focused on one *COI* clade containing populations sympatric with *P*. *kawadai*, and subdivided this clade into two analysis units (i.e., sympatric or allopatric with *P*. *kawadai*).

### 2.2 Morphological analysis

**FIGURE 2:**
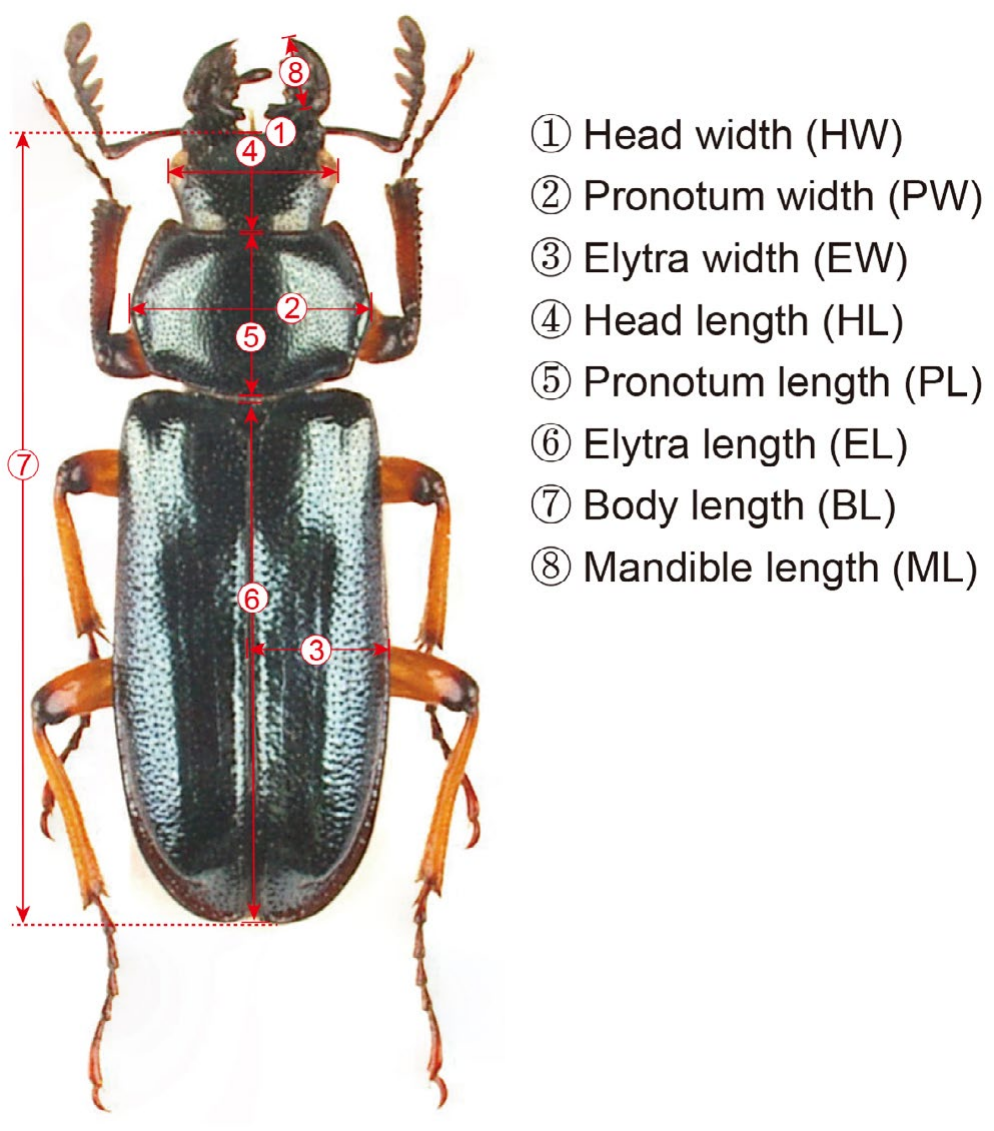
The eight investigated morphological traits investigated in this study. All traits were measured on the right side of the beetle's body, with the left side measured only when body parts were damaged or missing

For the morphological analysis, we assessed morphological external differentiation of *P*. *delicatulus* (central‐to‐northern Honshu) and *P*. *kawadai* specimens collected from 2005 to 2020, which were deposited in the Forest Zoology Laboratory of the University of Tokyo. We focused on external body size and shape using traits related to ecological specialization. Those selected morphological traits in this study are often associated with adaptation process as demonstrated by published literature (Hagge et al., 2021; Konuma et al., 2013; Okada & Miyatake, 2009). We firstly captured video images of specimens in dorsal view using a DP12 digital camera (Olympus, Tokyo) attached to an SZ10 stereoscopic microscope (Olympus). Then, we measured the eight morphological traits illustrated in Figure 2 from each habitus image using Photoshop software (Adobe, San Jose, CA) on a personal computer. We measured the trait lengths of adult beetles, including 213 specimens (111 males and 102 females) of *P*. *delicatulus* (23 sites for male and 24 sites for female) and 253 specimens (142 males and 113 females) of *P*. *kawadai* (26 sites for male and 22 sites for female).

To obtain a general view of the morphological differences among the populations, we first log‐transformed all trait measurements and performed a principal components analysis (PCA) using the procomp function in R v.3.6.3 (R Core Team, 2013) and visualized the results using “ggplot2” (Wickham & Wickham, 2007). To examine whether the two species differed in their morphological traits, we compared the principal component (PC) 1 and PC2 between two populations for each sex of each species. Then, we tested for body length (BL) differences between and within species and between the sexes using analysis of variance (ANOVA) and Tukey's HSD post hoc tests; BL was used as the measure for analysis, as it provides greater reproducibility than an axis derived from PCA (Barton et al., 2011).

**FIGURE 3:**
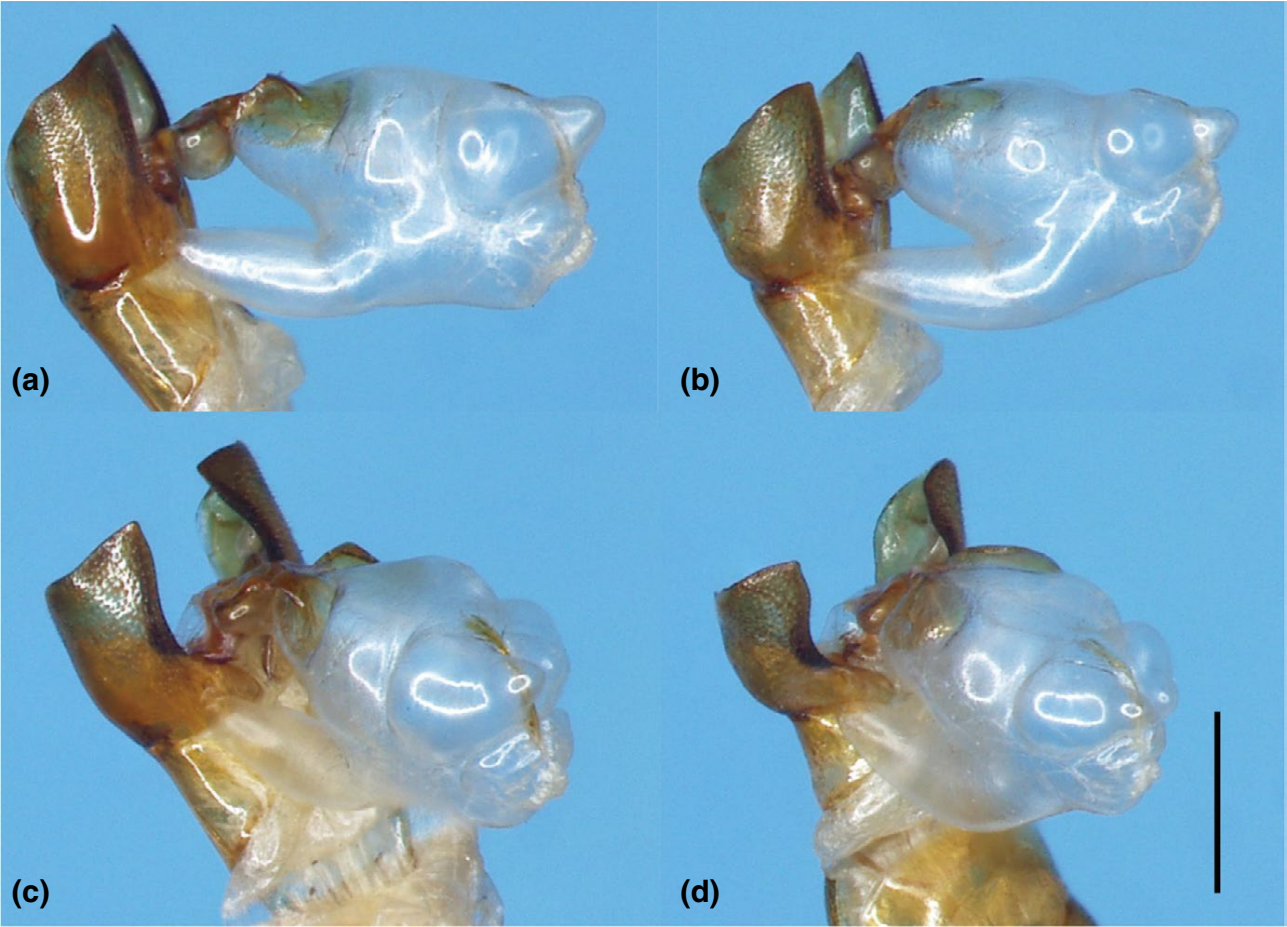
Male genital endophallus of *Platycerus delicatulus* (a, c) and *P*. *kawadai* (b, d). Membranous parts are endophalli. (a, b), Right lateral view; (c, d), right subdorsal view; scale, 1 mm

For genital morphology, although we observed little difference in endophallic structure between *P*. *delicatulus* and *P*. *kawadai* (Figure 3), which may be concerning for reproductive isolation, we found no difference among populations within each species. Quantitatively assessing the membranous part of the endophallus is difficult, so we did not consider genital morphological variation.

### 2.3 Environmental analysis

Environmental data were downloaded from the Worldclim database (v.1.4; http://www.worldclim.org; Hijmans et al., 2005) at a resolution of 30 arc seconds. A total of 99 occurrences of nonduplicated records (55 for *P*. *delicatulus* and 44 for *P*. *kawadai*) were obtained from field surveys and previous research (Zhang & Kubota, 2021). Next, we extracted 19 bioclimatic variables for each sampling location and tested multicollinearity among these variables. We excluded bioclimatic variables with a Pearson's correlation coefficient |*r*| > .8. Accordingly, we retained six climatic variables for subsequent analysis: Elevation (Ele), isothermality (Bio3), temperature seasonality (Bio4), mean temperature of wettest quarter (Bio8), annual precipitation (Bio12), and precipitation of coldest quarter (Bio19) (Tables 1 and 2).

**TABLE 1:** Summary of environmental variables used in this study

Code	Environmental variables	Unit
Ele	Elevation	m
Bio3	Isothermality	–
Bio4	Temperature seasonality	–
Bio8	Mean temperature of the wettest quarter	°C
Bio12	Annual precipitation	mm
Bio19	Precipitation of coldest quarter	mm

**TABLE 2:** Correlation for the environmental variables associated with *Platycerus* occurrence sites

	Bio3	Bio4	Bio8	Bio12	Bio19
Ele	0.75	−0.45	−0.11	0.21	−0.53
Bio3	1	−0.64	0.03	−0.06	−0.73
Bio4		1	−0.34	−0.33	0.28
Bio8			1	0.09	0.01
Bio12				1	0.37
Bio19					1

To quantify the environmental niches of *P*. *delicatulus* and *P*. *kawadai* populations, we used two statistical approaches. First, PCA was performed on the environmental variables using procomp function in R v.3.6.3 (R Core Team, 2013) and visualized using “ggplot2” (Wickham & Wickham, 2007). Second, we compared the environmental niche spaces of the species using n‐dimensional hypervolumes analyses (Hutchinson, 1957), which were conducted using the “hypervolume” R package (Blonder et al., 2018). We constructed the hypervolumes using the six retained variables for the major populations. All environmental variables were natural log‐transformed for analysis. All hypervolumes were created using the Gaussian kernel density estimator method with the default Silverman bandwidth estimator (Blonder et al., 2014, 2018). To compare hypervolumes among environmental variables, we quantified the pairwise overlap between populations, using the Jaccard and Sorensen similarity indexes following Blonder et al. (2018).

### 2.4 Correlations between morphology and environmental niche

We conducted Mantel tests and partial Mantel tests using the “vegan” R package to test correlation between the morphological and environmental distances of *P*. *delicatulus* and *P*. *kawadai* (Oksanen et al., 2013). Morphological distance was calculated as the Euclidean pairwise distance of BL between localities because BL is considered as an important trait for resource competition and reproductive interference (Okuzaki, 2021; Takami & Sota, 2007). Geographic distance was assessed as the Euclidean distance of latitude and longitude between localities. For environmental distance, we firstly scaled the six environmental variables prior to creating a distance matrix using scale function, because the environmental variables were all measured using different metrics that are not comparable to each other. Then, we calculated Euclidean pairwise distance of the environmental variables between sites using dist function (Oksanen et al., 2019). Finally, the significances between the geographic distance and morphological distance or between environmental and morphological distance were assessed by running 10,000 permutations. The partial Mantel test was used to determine whether morphological distance was correlated with environmental distance while controlling for the effect of geographic distance (Morpho, Env | Geo) based on Pearson correlation coefficients. Regression analysis was used to describe the relationship of the residual morphological values vs. residual geographic values and residual morphological values vs. residual environmental values for populations of each species.

## 3 RESULTS

### 3.1 Phylogenetic relationship between species

We sequenced 784 bp of the *COI* gene and 730–732 bp of the ITS region. These sequences were deposited in GenBank (DDBJ accession numbers: LC651809–LC651901 for the *COI* gene, and LC651902–LC651946 for the ITS region). The best‐fit models were GTR + I + G for *COI* and GTR + G for the ITS region.

**FIGURE 4:**
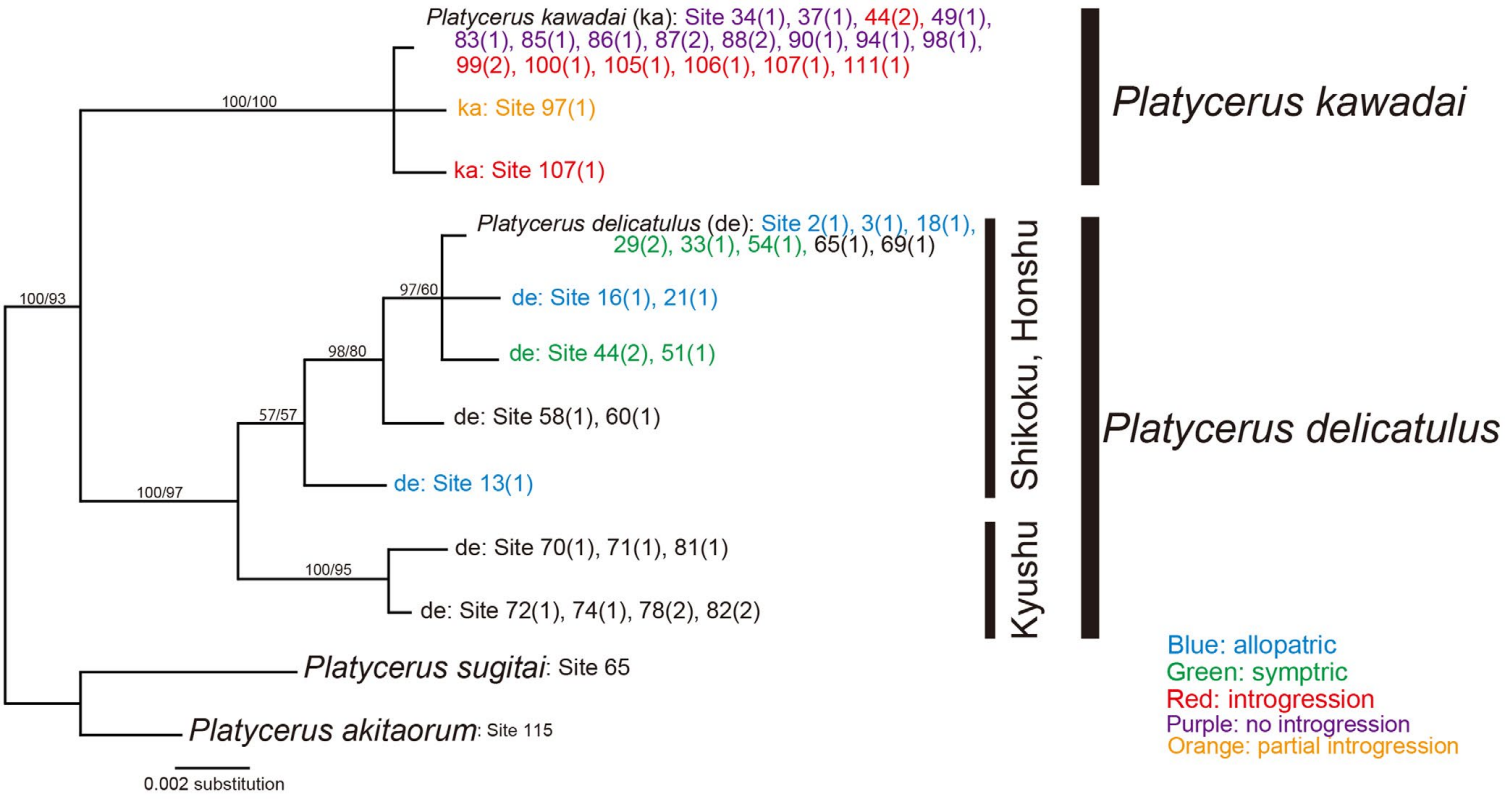
Consensus tree based on majority rule (>50%) of Bayesian inference (BI) tree for *Platycerus delicatulus* and *Platycerus kawadai* in Japan based on ITS sequences. *Platycerus akitaorum* and *Platycerus sugitai* were used as the outgroup. Operational taxonomic units indicate the combination of “species” and “site number (number of individuals sharing the same haplotype)”. Numbers near the branches indicate nodal support (posterior probability in the BI tree [> 50%] and bootstrap probability in the maximum‐likelihood (ML) tree [> 50%])

Based on the ITS region, *P*. *delicatulus* and *P*. *kawadai* constitute an independent distant monophyletic group, which aligned with the morphologically identified species units. *P*. *delicatulus* was subdivided into a Honshu and Shikoku population and a Kyushu population (Figure 4).

**FIGURE 5:**
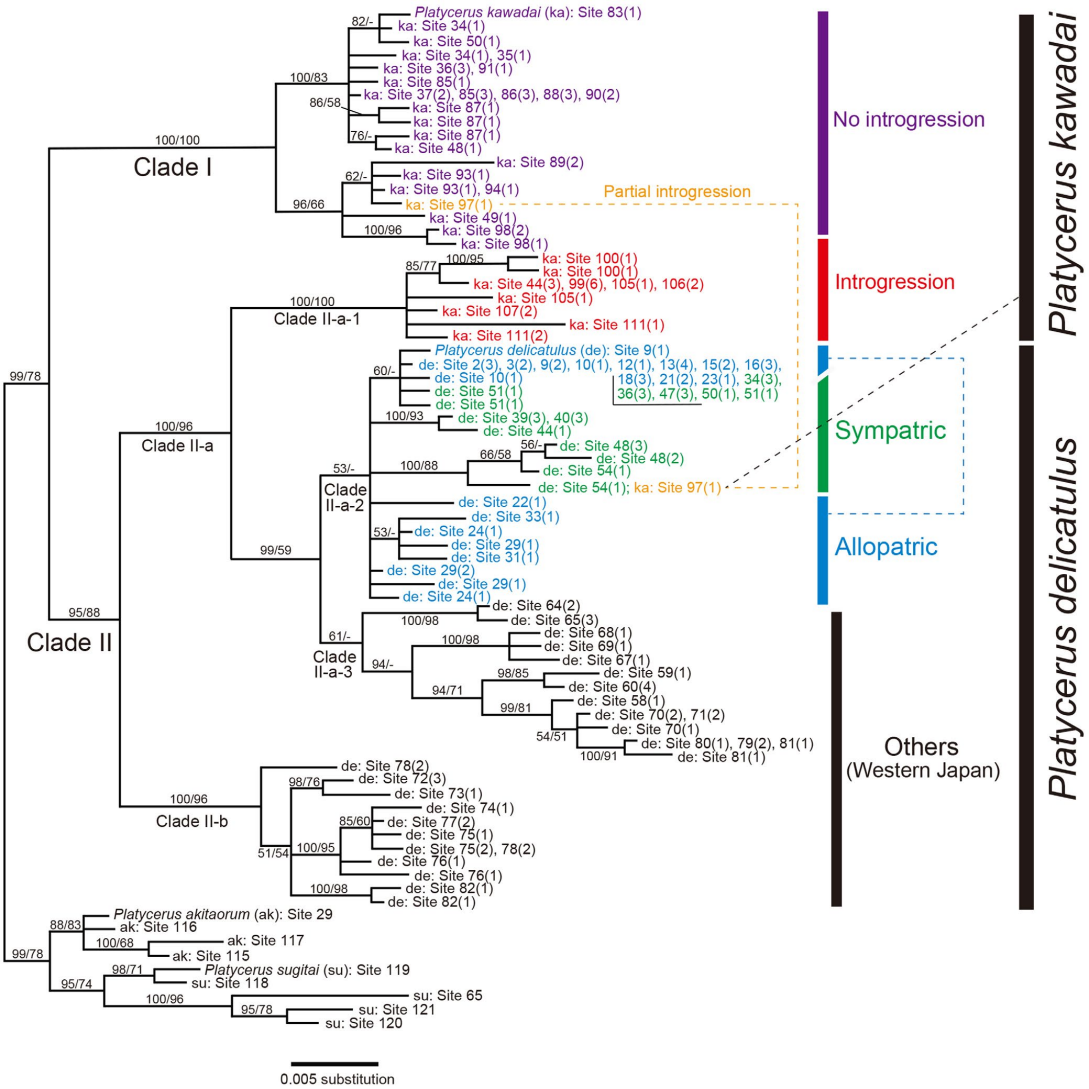
Consensus tree based on majority rule (>50%) of Bayesian inference (BI) tree for *Platycerus delicatulus* and *Platycerus kawadai* in Japan based on *COI* sequences. *Platycerus akitaorum* and *Platycerus sugitai* were used as the outgroup. Operational taxonomic units indicate the combination of “species” and “site number (number of individuals sharing the same haplotype).” Numbers near the branches indicate nodal support (posterior probability in the BI tree [> 50%] and bootstrap probability in the maximum‐likelihood (ML) tree [> 50%])

Two major clades were obtained based on the *COI* gene (Figure 5). Clade I was composed of entirely of *P*. *kawadai*, whereas Clade II contained both species. Clade II‐a‐1 composed of *P*. *kawadai* based on morphology and was assumed to contain the offspring of a population that receive mitochondrial genes from *P*. *delicatulus* via the introgressive hybridization. Clades II‐a‐2, II‐a‐3, and II‐b were composed mainly of *P*. *delicatulus*. However, a male *P*. *kawadai* collected at Site 97 was in Clade II‐a‐2, whereas another individual from that site belonged to Clade I (Figure 5).

**FIGURE 6:**
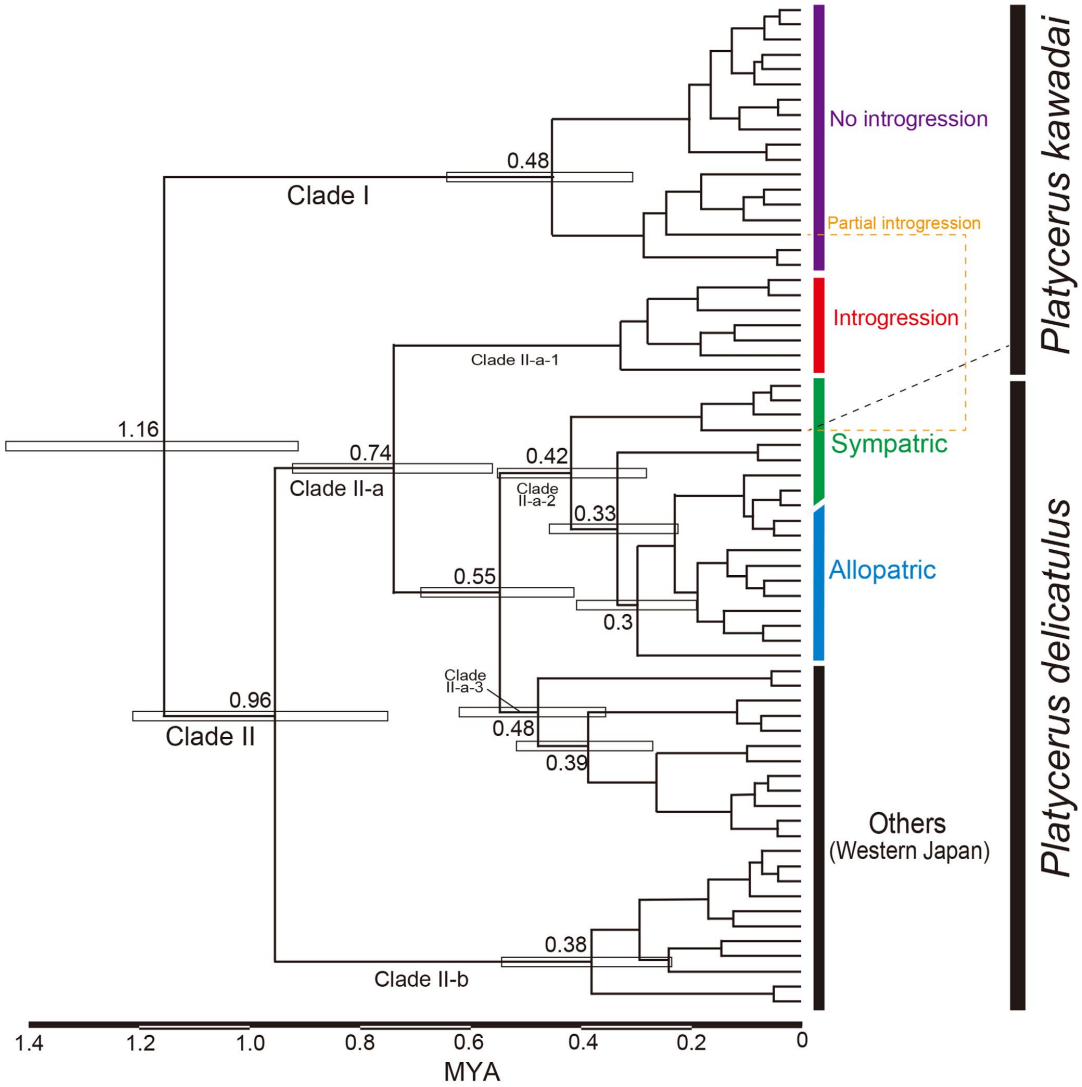
Divergence time estimates of *Platycerus delicatulus* and *Platycerus kawadai* in a time‐calibrated tree based on the *COI* gene. Numbers and squares near the divergence points indicate divergence times and their 95% confidence intervals, respectively

The divergence times of *P*. *delicatulus* and *P*. *kawadai* populations were estimated based on the *COI* gene (Figure 6). The estimated divergence time between Clades I and II (representing the speciation between *P*. *delicatulus* and *P*. *kawadai*) was 1.16 Mya. Clade II was subdivided into Clade II‐a (generally, *P*. *delicatulus*: Honshu, Shikoku, and northern Kyushu) and Clade II‐b (*P*. *delicatulus*: southern Kyushu) at 0.96 Mya. The introgressive hybridization that was the origin of Clade II‐a‐1 occurred approximately 0.74 Mya. In the recent past, an introgressive hybridization occurred at Site 97 (Figures 5 and 6).

For subsequent analyses, in the context of the interspecific relationship and intraspecific divergence, we subdivided *P*. *kawadai* populations into two analysis units: “No introgression” population (Clade I) and “Introgression” population (Clade II‐a‐1) based on the molecular results. In this classification, we excluded the population at Site 97 with a *P*. *kawadai* sample exhibiting the introgression type for *COI* gene from *P*. *delicatulus*. It is a very rare case because all other samples from the same mountain range (Akaishi Mountains) including Site 97 exhibited no introgression type. Sites at which no genetic samples were collected were assigned to the category of the closest site at which genetic samples were collected. We subdivided *P*. *delicatulus* populations belonging to Clade II‐a‐2 into “Sympatric” population and “Allopatric” population. Sympatric population range covers whole range of *P*. *kawadai*, whereas both species cannot be always collected at the same site (Figure 1, Appendix 1). In the following part, we examined the morphological differentiation among these analysis units of two species.

### 3.2 Morphological differentiation

**FIGURE 7:**
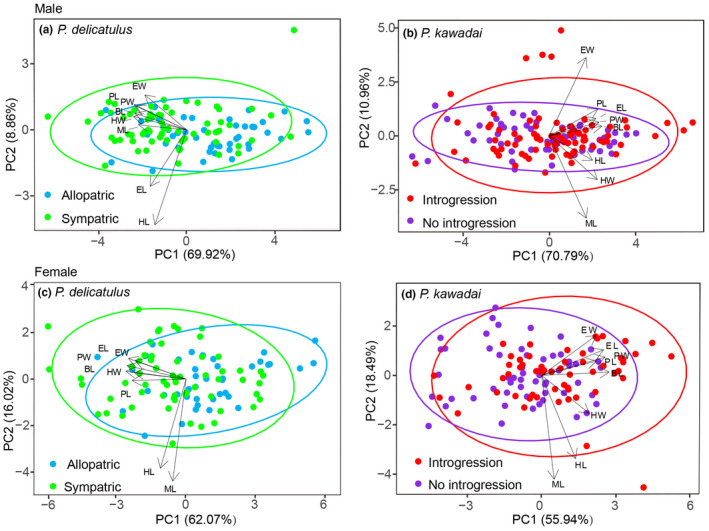
Principal component analysis plots of morphological data showing differentiation between populations of *Platycerus delicatulus* and *Platycerus kawadai*. Ellipses represent the 95% confidence intervals

**FIGURE 8:**
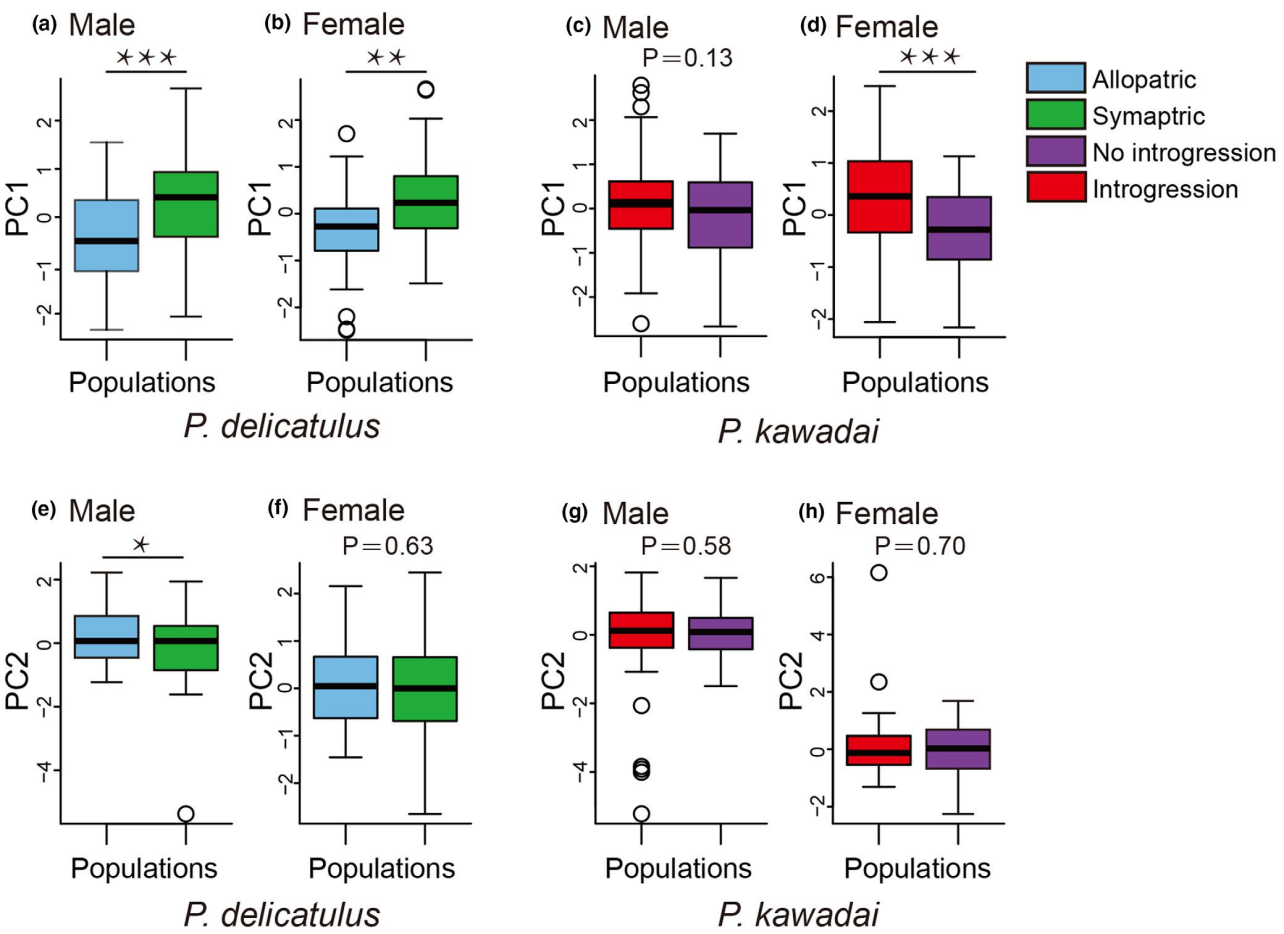
Morphological differentiation between populations along the first two principal components (PC1, a–d; PC2, e–h) for *Platycerus delicatulus* male (a, e) and female (b, f) individuals, and *P*. *kawadai* male (c, g) and female (d, h) individuals. Student's *t*‐test results are also shown. *, *p* < .05; **, *p* < .01; ***, *p* < .001

**FIGURE 9:**
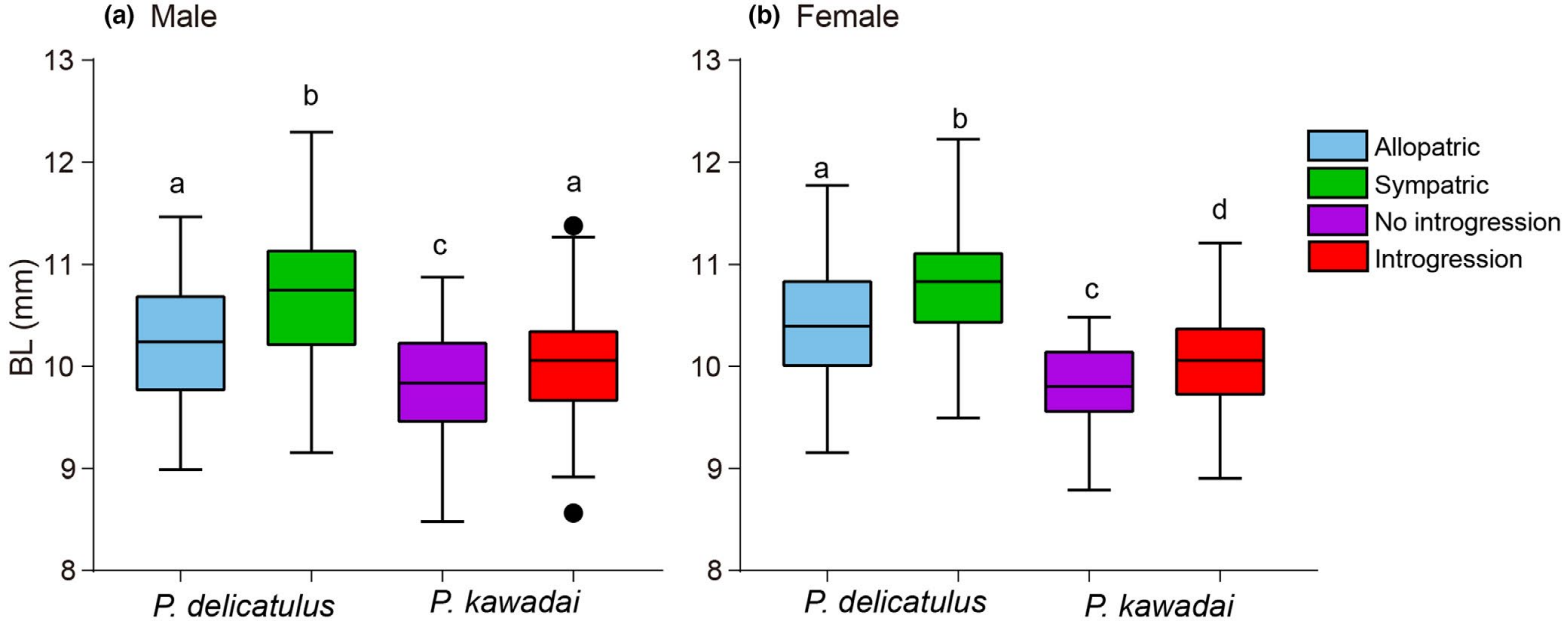
Morphological differentiation between populations with respect to variations in body length (BL) for both male (a) and female (b) individuals. Analysis of variance (ANOVA) results are also shown. Different letters indicate significant differences between populations (Tukey's test: *p* < .05)

Examinations of morphological variation in eight traits by PCA indicated differentiation between Allopatric and Sympatric populations of *P*. *delicatulus*, as well as between No introgression and Introgression populations of *P*. *kawadai* mainly along the PC1 axis (Figure 7). Specifically, male and female populations of *P*. *delicatulus* were mainly discriminated by the first principal component (PC1), which explained 69.92% and 62.07% of the variance, respectively. For *P*. *kawadai*, PC1 explained 70.79% and 55.94% of the total variance for male and female, respectively. The significant difference between the populations in PC2 was detected only for *P*. *delicatulus* males (Figure 8). In this case, the eigenvalue of PC2 was 0.71 and the highest loading score for PC2 was 0.68 of head length (HL) (Table 3). PC2 and HL could not sufficiently explain the morphological differentiation between the populations. On the other hand, the significant difference between populations in PC1 was detected for most studied species and sexes except for *P*. *kawadai* males (Figure 8). BL exhibited the highest loading scores on the first axis PC1 (0.95–0.97) in both species and sexes (Tables 3 and 4). Additionally, BL showed a significant level of differentiation between Allopatric and Sympatric populations of *P*. *delicatulus*, as well as between No introgression and Introgression populations of *P*. *kawadai* for both male and female individuals (*p* < .001, ANOVA; Figure 9), but we found no significant differentiation in BL between Allopatric populations of *P*. *delicatulus* and Introgression populations of *P*. *kawadai* for males (Figure 9a). On the other hand, female BL varied significantly between the two species (Figure 9b). Sympatric population of *P*. *delicatulus* and No introgression population of *P*. *kawadai* showed the highest and lowest value, respectively (Figure 9).

**TABLE 3:** Principal component analysis (PCA) loading scores for morphological traits used to evaluate the morphological differentiation for males of *Platycerus delicatulus*

Morphological traits	Male		Female	
	PC1	PC2	PC1	PC2
Head width (HW)	0.93	−0.10	0.83	−0.05
Pronotum width (PW)	0.94	−0.16	0.94	−0.11
Elytra width (EW)	0.75	−0.22	0.81	−0.15
Head length (HL)	0.63	0.68	0.41	0.72
Pronotum length (PL)	0.91	−0.15	0.86	0.01
Elytra length (EL)	0.65	0.36	0.92	−0.17
Body length (BL)	**0.95**	−0.09	**0.96**	−0.06
Mandible length (ML)	0.86	−0.03	0.21	0.83
Eigenvalue	5.59	0.71	4.97	1.28
% of variance	69.92	8.86	62.07	16.02

Note

The trait that contributed the most is highlighted in bold on PC1.

**TABLE 4:** Principal component analysis (PCA) loading scores for morphological traits used to evaluate the morphological differentiation of *Platycerus kawadai*

Morphological traits	Male		Female	
	PC1	PC2	PC1	PC2
Head width (HW)	0.88	0.32	0.68	0.31
Pronotum width (PW)	0.92	−0.10	0.91	−0.15
Elytra width (EW)	0.68	−0.57	0.78	−0.33
Head length (HL)	0.80	0.18	0.48	0.68
Pronotum length (PL)	0.86	−0.13	0.78	−0.12
Elytra length (EL)	0.89	−0.19	0.88	−0.21
Body length (BL)	**0.97**	−0.07	**0.96**	−0.03
Mandible length (ML)	0.68	0.60	0.18	0.85
Eigenvalue	5.66	0.87	4.47	1.48
% of variance	70.79	10.96	55.94	18.49

Note

The trait that contributed the most is highlighted in bold on PC1.

### 3.3 Environmental niche

**FIGURE 10:**
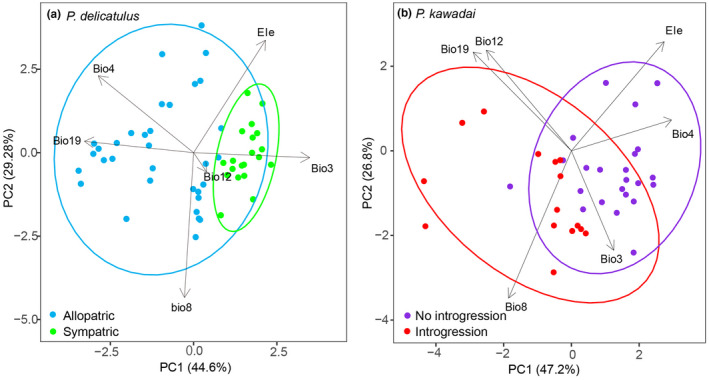
Principal component analysis (PCA) plots of environmental variables (see Table 1) showing differentiation between populations of *Platycerus delicatulus* (a) and *Platycerus kawadai* (b). Ellipses represent the 95% confidence intervals

For *P*. *delicatulus*, we found the PCA results suggested that Sympatric population had a narrower environmental space than that of Allopatric population, especially in terms of elevation, temperature seasonality (Bio4), and mean temperature in wettest quarter (Bio8) (Figure 10a; Table 5). Two principal components (PC) explained 44.6% (PC1) and 29.28% (PC2) of the variation between populations of *P*. *delicatulus*. For *P*. *kawadai*, two primary principal components (PC) accounted for 47.2% (PC1) and 26.8% (PC2) of the total variance (Figure 10b). No introgression population exhibited higher temperature seasonality and lower mean temperature of wettest quarter, favoring less precipitation (Bio12 and Bio19) and a wider elevation compared with the Introgression population of *P*. *kawadai* (Table 5).

**TABLE 5:** Principal component analysis (PCA) loading scores for environmental predictors used to evaluate the environmental niche for *Platycerus delicatulus*

Environmental predictors	*P. delicatulus*		*P. kawadai*	
	PC1	PC2	PC1	PC2
Elevation (Ele)	−0.59	0.74	−0.77	0.55
Isothermality (Bio3)	**0.95**	−0.03	−0.35	−0.50
Temperature seasonality (Bio4)	0.78	0.51	**−0.83**	0.15
Mean temperature of the wettest quarter (Bio8)	0.07	**−0.96**	0.52	**−0.74**
Annual precipitation (Bio12)	−0.11	−0.13	0.70	0.50
Precipitation of coldest quarter (Bio19)	0.89	0.08	0.81	0.49
Eigenvalues	2.68	1.76	2.83	1.60
% of variance	44.60	29.28	47.20	26.80

Note

The predictor that contributed the most is highlighted in bold on each axis.

**FIGURE 11:**
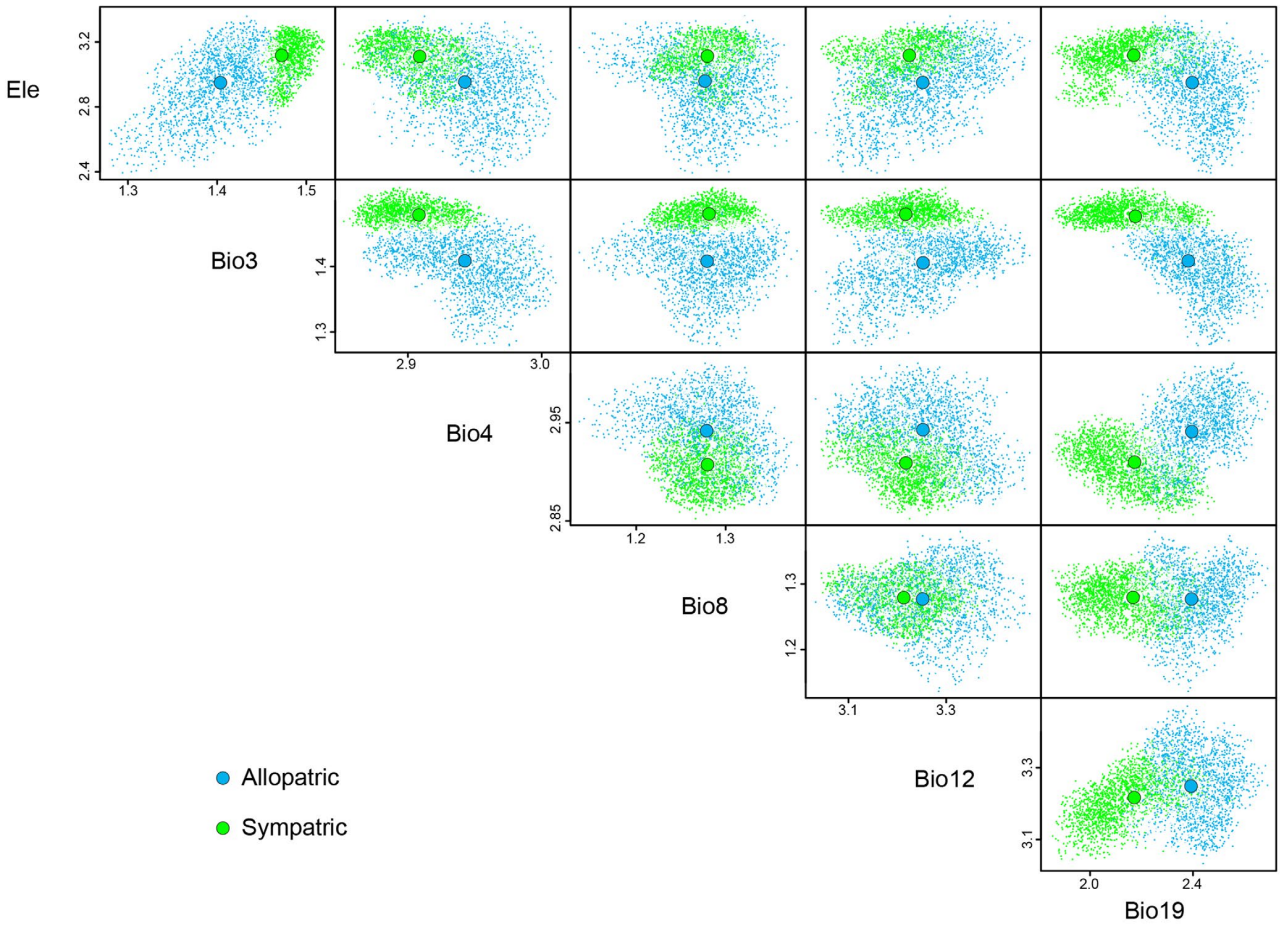
Hypervolumes obtained from multidimensional kernel density estimation of the studied population (Allopatric and Sympatric population) of *Platycerus delicatulus* based on weakly correlated environmental variables. The larger colored dots represent species centroids

**FIGURE 12:**
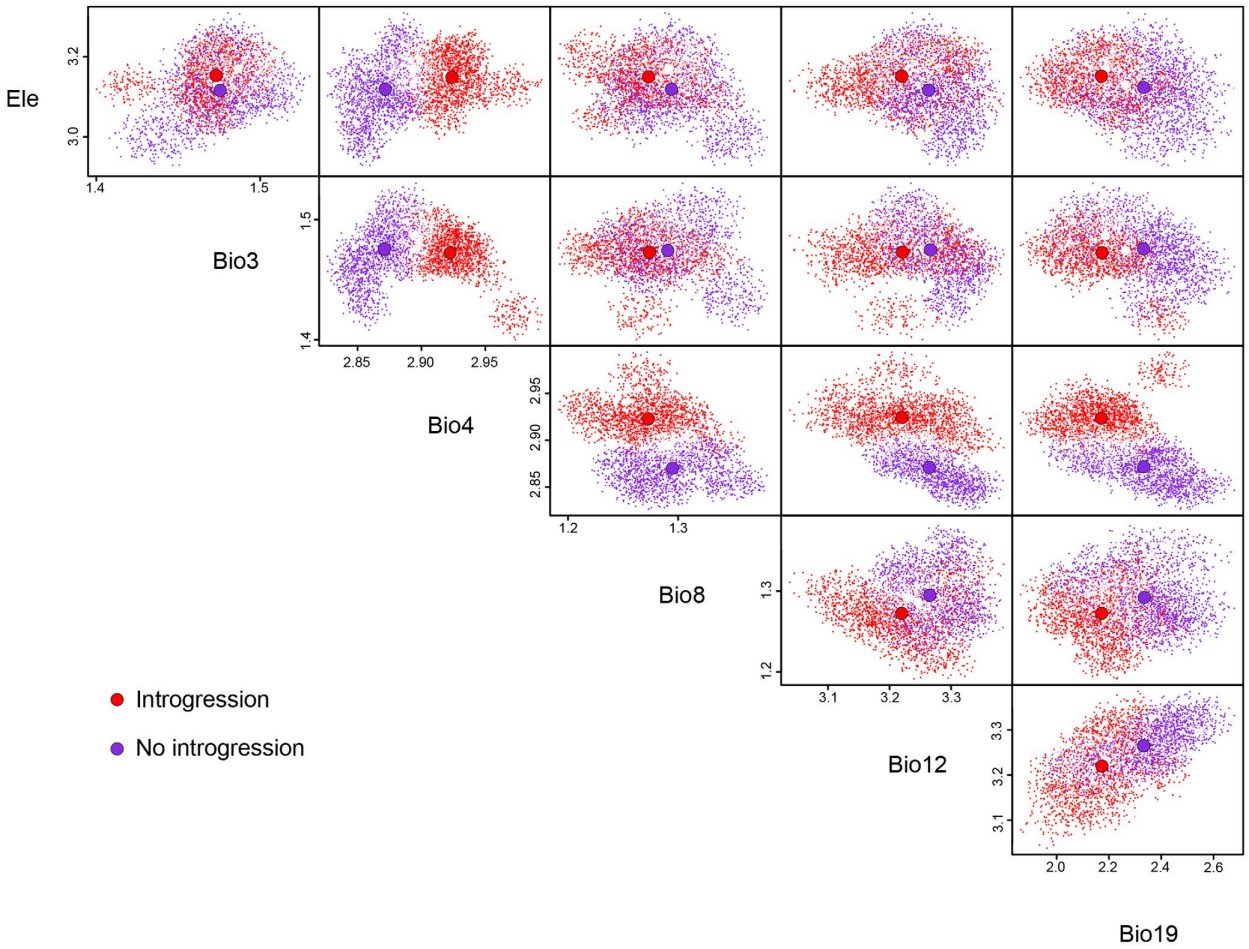
Hypervolumes obtained from multidimensional kernel density estimation of the studied population (Allopatric and Sympatric population) of *Platycerus kawadai* based on weakly correlated environmental variables. The larger colored dots represent species centroids

The multidimensional variations in the environmental space of both species are shown as niche hypervolumes in Figures 11 and 12, illustrating that the populations occupied different ecological spaces with relatively little overlap. For *P*. *delicatulus*, the niche hypervolume was much greater for the Allopatirc population than for the Sympatric population, and they overlapped slightly (Sørensen similarity = 0.057, Jaccard similarity = 0.029; Figure 11). For *P*. *kawadai*, the Sørensen and Jaccard similarity index values of the hypervolumes were 0.135 and 0.072 in No introgression and Introgression populations, respectively. Generally, Bio4 did not overlapped between the populations (Figure 12).

### 3.4 Correlation between morphological and environmental niche

**FIGURE 13:**
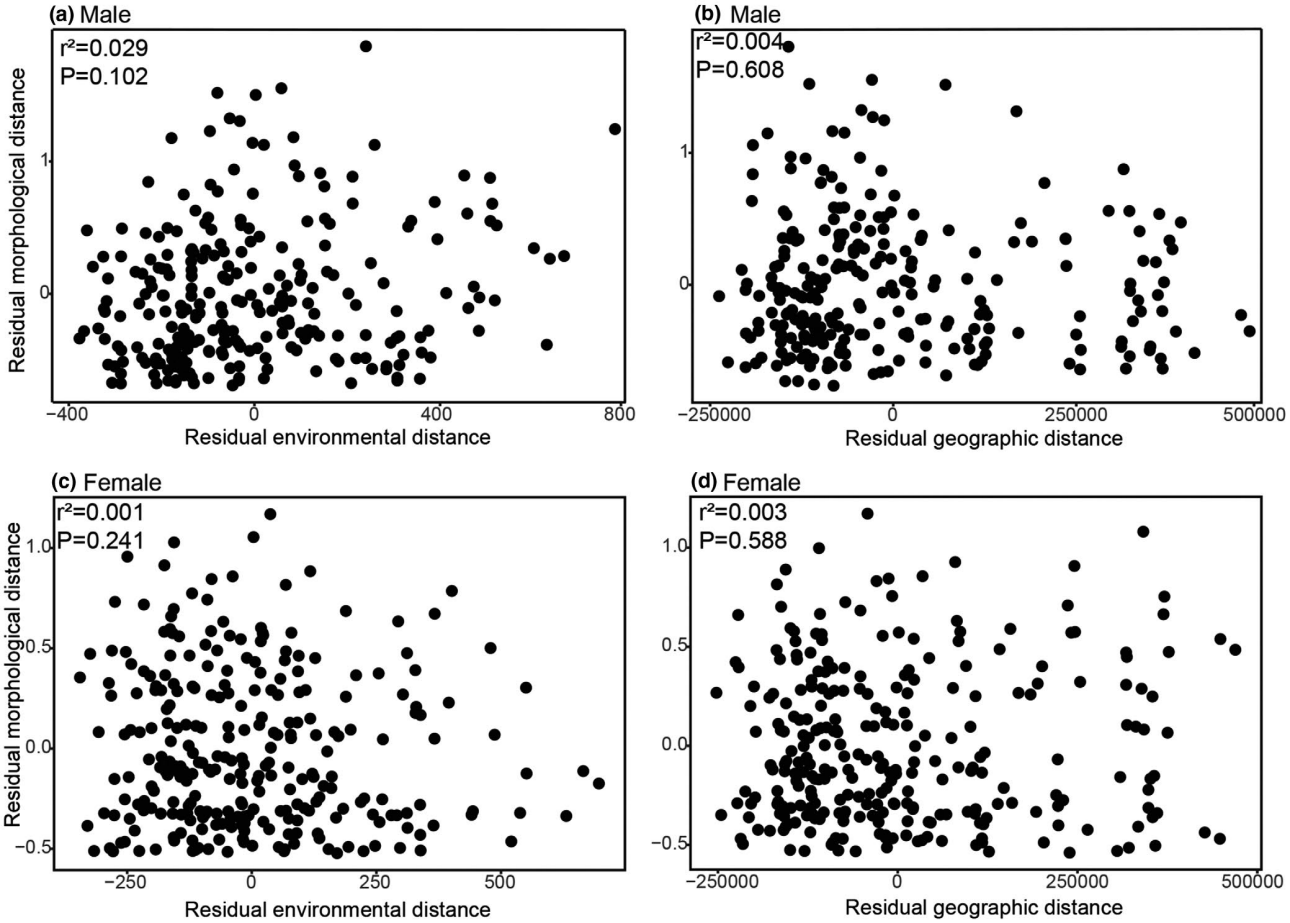
Partial regression plots illustrating the relationship between morphological distance and the environmental distance controlling geographic distance (a and c), and between morphological distance and geographic distance controlling for environmental distance (b and d) for male and female of *Platycerus delicatulus*, respectively

**FIGURE 14:**
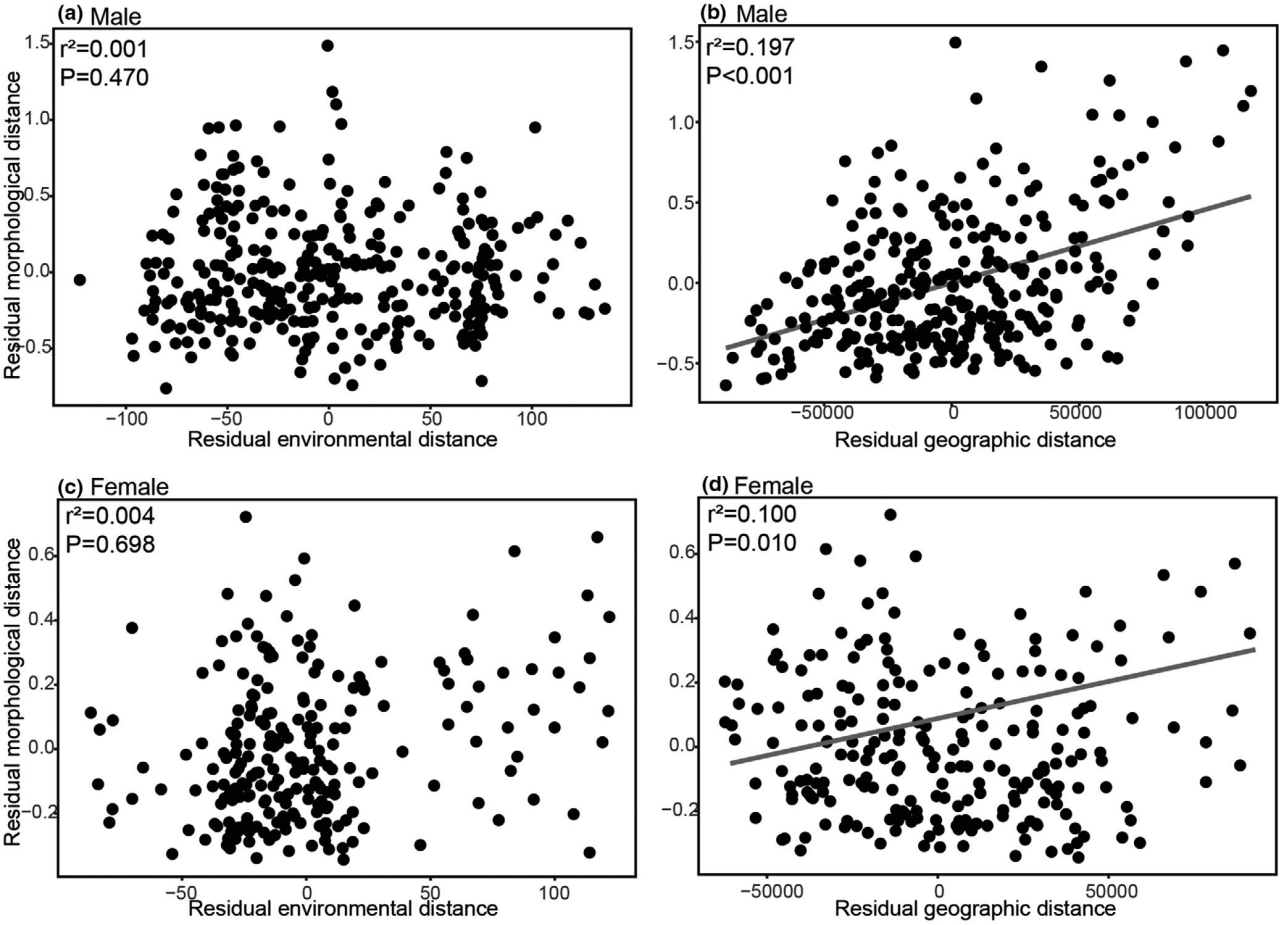
Partial regression plots illustrating the relationship between morphological distance and the environmental distance controlling geographic distance (a and c), and between morphological distance and geographic distance controlling for environmental distance (b and d) for male and female of *Platycerus kawadai*, respectively. Lines represent significant regressions of the residuals

For *P*. *delicatulus*, simple Mantel tests showed that the morphological distance between populations was not significantly correlated with environmental (male, *p* = .104; female, *p* = .283) or geographic distances (male, *p* = .119; female, *p* = .315) (Table 6). Morphological distance was not related with environmental distance after controlling for the effect of geographic distance (male, *p* = .102; female, *p* = .241, Figure 13a,c) and with geographic distance after controlling for environmental distance (male, *p* = .608; female, *p* = .588, Figure 13b,d) based on the partial Mantel test results. On the other hand, for *P*. *kawadai*, morphological distance was significantly correlated with the environmental (male, *p* = .005; female, *p* = .03) and geographic distances (male, *p* < .001; female, *p* = .003) (Table 6). Morphological distances were not significantly correlated with environmental distances after controlling for geographic distances using partial Mantel tests for *P*. *kawadai* (male, *p* = .470; female, *p* = .698, Figure 14a,c), however, morphological distance was significantly correlated with geographic distances after controlling for environmental distance in the same manner (male, *p *< .001; female, *p* = .010, Figure 14b,d).

**TABLE 6:** Single and partial Mantel test results based on morphological, environmental, and geographic distances between occurrence sites of *Platycerus delicatulus* and *P*. *kawadai*

Comparison	Sex	*P. delicatulus*		*P. kawadai*	
		*r*	*p*‐Value	*r*	*p*‐Value
Single Mantel tests					
Morphological and environmental	Males	.160	.104	.170	.**006**
	Females	.048	.283	.331	.**007**
Morphological and geographic	Males	.062	.119	.469	**<.001**
	Females	.024	.315	.249	.**003**
Partial Mantel tests					
Morphological and environmental | geographic	Males	.170	.102	.009	.470
	Females	.058	.241	.059	.698
Morphological and geographic | environmental	Males	.059	.608	.443	**<.001**
	Females	.032	.588	.316	.**010**

Note

Bold values denote statistical significance at the *p* < .05 level.

## 4 DISCUSSION

### 4.1 Phylogeographic history of the two related species

The genetic sample collection sites of the two species cover almost their entire distribution ranges (Appendix 2). Phylogenetic analyses based on the ITS region suggested that *P*. *delicatulus* and *P*. *kawadai* are each essentially monophyletic (Figure 4). This result aligns with the phylogenetic results of their yeast symbionts (Kubota et al., 2020). Since the ancestral branches of *P*. *delicatulus* diverged in western Japan, it is likely that the two species were separated and speciated in western (*P*. *delicatulus*) and central (*P*. *kawadai*) Japan approximately 1.16 Mya (Figures 5 and 6). Following that speciation event, *P*. *delicatulus* was separated into two clades (Clade II‐a: Honshu, Shikoku, and northern Kyushu; and Clade II‐b: southern Kyushu in *COI*) approximately 0.96 Mya. The Clade II‐a population of *P*. *delicatulus* expanded eastward, and hybridized with *P*. *kawadai* after 0.74 Mya, which resulted in portion of *P*. *kawadai* forming a clade (Clade II‐a‐1: Introgression population) nested within the *P*. *delicatulus* clade (Clade II). Since then, introgressive hybridization appears to have occurred very rarely between the two species (Figures 5 and 6). Moreover, in terms of the direction of introgression, morphological similarity may have resulted in a relatively higher probability of introgression from *P*. *delicatulus* to *P*. *kawadai* than in the reverse direction. *P*. *delicatulus* females and *P*. *kawadai* males may occasionally mate with each other because females of *P*. *delicatulus* have a larger body size than *P*. *kawadai* and mitochondrial genes are maternally inherited only. Based on our observation, males of *Platycerus* species always try to mate immediately with any female during the reproductive season. When there is a chance of heterospecific mating, interspecific differences in body size and genitalia size may work as premating and mechanical isolation mechanisms, respectively (Kubota & Sota, 1998; Takami & Sota, 2007; Okuzaki, 2021). A similar phylogeographic pattern has been documented in other beetles (Kosuda et al., 2016; Takami et al., 2007; Zhang & Sota, 2007).

These results indicated that No introgression population and Introgression population of *P*. *kawadai* differed mainly in terms of *COI*, but they cannot be distinguished using ITS sequences. Possible explanations for the mitochondrial–nuclear discordance could be associated with sex‐biased dispersal, mating, and offspring production (Bonnet et al., 2017). Genetic drift is ubiquitous in populations and can interact with many of the above processes to increase discordance between mitochondrial and nuclear genes (Toews & Brelsford, 2012). But it is difficult to explain the essential topological difference between the *COI* and ITS phylogenies just for these reasons. Another possible evolutionary scenario for such a discordance is the incomplete lineage sorting following the ancestral polymorphism of mitochondrial gene (Funk & Omland, 2003). However, it is unlikely that the ancestor of *P*. *kawadai* had possessed both mitochondrial Clades I and II‐a‐1 because Clade II‐a‐1 had occurred in a *P*. *delicatulus* type subclade (Clade II‐a) after initial geographical differentiation within *P*. *delicatulus*. An alternative and more likely scenario is historical mitochondrial introgression following the range expansion of these species. Because Clade II‐a‐1 was diverged from a *P*. *delicatulus* type clade around 0.74 Mya, the replacement by an introgressive clade seems to be very rare and only one replacement is recognized.

### 4.2 Factors affecting morphological differences among *Platycerus* populations within species

In this study, we constructed intraspecific analysis units of two *Platycerus* species based on interspecific ranges and evolutionary dynamics, and then evaluated the factors affecting the morphological differences within each species. Among the eight morphological traits shown in Figure 3, BL was the most effective variable for explaining morphological variation (Figure 7). Meanwhile, the results of the n‐dimensional hypervolume analysis revealed environmental heterogeneity among populations. We tested whether the morphological variation across populations was better explained by geographic distance with dispersal or by environmental filtering for studied species.

For *P*. *delicatulus*, the morphological (BL) distance among collection sites was not correlated with environmental factors or with geographic distance, and therefore these factors could not explain the morphological divergence between Allopatric and Sympatric populations. The latter population is larger than the former, and likely arose via character displacement against *P*. *kawadai* (Figure 9). As *P*. *delicatulus* and *P*. *kawadai* are capable of mating, the putative character displacement may be caused by reproductive interference other than the resource competition. Overall, our results suggest that interspecific interaction has played a major role in driving the morphological differentiation of *P*. *delicatulus* populations.

For *P*. *kawadai*, morphological distance was correlated with geographic distance after controlling for environmental distance (Table 6). This result suggests that geographic distance (i.e., low dispersal ability) might have led to morphological differentiation. Therefore, dispersal is assumed to drive the morphological diversification of populations. Meanwhile, dispersal ability could influence range limits and gene flow among populations, which may be associated with niche differentiation. In addition, previous studies showed that morphological adaptation to local ecology can also have resulted from phenotypic plasticity or from genetic differences among populations (Borokini et al., 2021; Ghalambor et al., 2007; Kunz et al., 2022; Price et al., 2003; Schmid & Guillaume, 2017). Although phenotypic plasticity has been documented in response to variations in multiple environmental variables (Chevin & Lande, 2015; Gratani, 2014; Lande, 2009; Wang et al., 2021), we found morphological distance was not correlated with environmental distance after controlling for geographic distance (Table 6). Thus, environmental factors are unlikely to be responsible for the observed morphological differentiation in *P*. *kawadai*. However, we cannot exclude the possibility that genetic divergence, such as that achieved via genetic drift and intra‐ and interspecific gene flow, promoted the morphological divergence. Further studies are required to verify whether this possibility would explain the morphological differentiation among populations of *P*. *kawadai*.

Populations often experience different environmental conditions, leading to the evolution of different phenotypes to maximize fitness (Freudiger et al., 2021; Jones et al., 2021). Most studies have shown that body size is affected by environmental filtering and food availability, which exhibit trade‐off relationships (Dmitriew, 2011; Konuma et al., 2011; Runemark et al., 2015). Our results showed that intraspecific morphological variations in *P*. *delicatulus* and *P*. *kawadai* were related to interspecific interaction and geographic distance, respectively. These results indicated divergence between populations in directions of morphological variation and provided significant insights into species adaptation processes.

In conclusion, we integrated morphological, environmental, and molecular data across the geographic ranges of two species to investigate the ecological–evolutionary processes that may drive divergence processes among populations and across geography. We found that morphological and ecological niche differentiation within species may be driven by interspecific interaction, as well as dispersal ability. These differentiations may associate with specialization for habitat preference. Our results elucidate ecological process across species’ distributions through adaptation and plasticity in natural systems. Evidence of divergence between populations provides a useful reference for conservation strategies to enhance potential for adaptive response to the challenging climate changes.

## CONFLICT OF INTERESTS

The authors declare no conflict of interest.

## AUTHOR CONTRIBUTIONS

**Sheng‐Nan Zhang:** Conceptualization (lead); Data curation (equal); Formal analysis (lead); Investigation (equal); Methodology (lead); Writing – original draft (lead); Writing – review & editing (supporting). **Kôhei Kubota:** Conceptualization (supporting); Data curation (equal); Formal analysis (supporting); Funding acquisition (lead); Investigation (equal); Methodology (supporting); Project administration (lead); Resources (lead); Supervision (lead); Writing – original draft (supporting); Writing – review & editing (lead).

## Data Availability

Sequence data are available at the DDBJ database under accession numbers LC651809–LC651901 for the COI gene, and LC651902–LC651946 for the ITS region, https://www.ddbj.nig.ac.jp/ddbj/index‐e.html.

## APPENDIX 1 Occurrence records of Platycerus examined

**Table:**

Species	Analysis unit	Elevation (m)	Latitude (°)	(°)	Site No.
*P. delicatulus*	Allopatric	370	41.15	140.38	1
*P. delicatulus*	Allopatric	700	40.50	140.83	2
*P. delicatulus*	Allopatric	410	40.49	140.93	3
*P. delicatulus*	Allopatric	430	40.51	140.97	4
*P. delicatulus*	Allopatric	1000	38.52	139.73	5
*P. delicatulus*	Allopatric	700	38.28	140.46	6
*P. delicatulus*	Allopatric	640	38.48	140.01	7
*P. delicatulus*	Allopatric	740	38.53	139.96	8
*P. delicatulus*	Allopatric	460	38.21	139.85	9
*P. delicatulus*	Allopatric	880	38.14	140.51	10
*P. delicatulus*	Allopatric	1000	37.06	139.48	11
*P. delicatulus*	Allopatric	800	37.09	139.59	12
*P. delicatulus*	Allopatric	960	36.93	140.28	13
*P. delicatulus*	Allopatric	1300	36.87	139.40	14
*P. delicatulus*	Allopatric	1280	36.75	139.44	15
*P. delicatulus*	Allopatric	900	36.75	138.83	16
*P. delicatulus*	Allopatric	1220	36.67	138.67	17
*P. delicatulus*	Allopatric	1130	36.48	138.88	18
*P. delicatulus*	Allopatric	1300	36.77	138.82	19
*P. delicatulus*	Allopatric	1100	36.85	137.83	20
*P. delicatulus*	Allopatric	1250	36.38	137.75	21
*P. delicatulus*	Allopatric	1320	36.14	136.73	22
*P. delicatulus*	Allopatric	1030	35.52	136.41	23
*P. delicatulus*	Allopatric	550	34.46	136.24	24
*P. delicatulus*	Allopatric	1410	34.38	136.09	25
*P. delicatulus*	Allopatric	1300	34.35	136.21	26
*P. delicatulus*	Allopatric	1200	34.32	136.20	27
*P. delicatulus*	Allopatric	1200	34.21	136.12	28
*P. delicatulus*	Allopatric	1520	34.19	136.10	29
*P. delicatulus*	Allopatric	1150	34.22	135.98	30
*P. delicatulus*	Allopatric	1250	33.90	135.65	31
*P. delicatulus*	Allopatric	1250	34.15	135.65	32
*P. delicatulus*	Allopatric	690	35.35	135.74	33
*P. delicatulus*	Sympatric	1260	36.41	138.67	34
*P. delicatulus*	Sympatric	1400	36.20	138.64	35
*P. delicatulus*	Sympatric	1300	35.94	138.80	36
*P. delicatulus*	Sympatric	1100	35.91	138.82	37
*P. delicatulus*	Sympatric	780	35.92	138.84	38
*P. delicatulus*	Sympatric	1420	35.85	138.98	39
*P. delicatulus*	Sympatric	1450	35.74	139.02	40
*P. delicatulus*	Sympatric	1210	35.48	139.17	41
*P. delicatulus*	Sympatric	1567	35.47	139.16	42
*P. delicatulus*	Sympatric	1587	35.51	139.07	43
*P. delicatulus*	Sympatric	1400	35.51	139.05	44
*P. delicatulus*	Sympatric	1570	35.69	138.88	45
*P. delicatulus*	Sympatric	1100	35.78	138.77	46
*P. delicatulus*	Sympatric	1200	35.86	138.56	47
*P. delicatulus*	Sympatric	1550	35.38	138.53	48
*P. delicatulus*	Sympatric	1420	35.32	138.36	49
*P. delicatulus*	Sympatric	1480	36.90	138.49	50
*P. delicatulus*	Sympatric	1240	36.41	138.60	51
*P. delicatulus*	Sympatric	1550	35.39	137.99	52
*P. delicatulus*	Sympatric	1620	35.13	138.04	53
*P. delicatulus*	Sympatric	1180	35.23	137.99	54
*P. delicatulus*	Sympatric	1260	35.12	137.90	55
*P. delicatulus*	(Others)	1050	35.25	134.39	56
*P. delicatulus*	(Others)	1100	35.19	133.82	57
*P. delicatulus*	(Others)	970	35.35	133.54	58
*P. delicatulus*	(Others)	1100	34.69	132.19	59
*P. delicatulus*	(Others)	1080	34.50	132.13	60
*P. delicatulus*	(Others)	1220	33.92	134.34	61
*P. delicatulus*	(Others)	1120	33.91	134.29	62
*P. delicatulus*	(Others)	1030	33.92	134.29	63
*P. delicatulus*	(Others)	1220	33.88	134.11	64
*P. delicatulus*	(Others)	1320	33.87	134.09	65
*P. delicatulus*	(Others)	1140	33.94	132.94	66
*P. delicatulus*	(Others)	1430	33.75	133.15	67
*P. delicatulus*	(Others)	1480	33.48	133.02	68
*P. delicatulus*	(Others)	1150	33.19	132.61	69
*P. delicatulus*	(Others)	960	33.48	130.93	70
*P. delicatulus*	(Others)	740	33.46	130.91	71
*P. delicatulus*	(Others)	1100	33.28	131.40	72
*P. delicatulus*	(Others)	880	33.12	131.29	73
*P. delicatulus*	(Others)	1620	32.58	131.11	74
*P. delicatulus*	(Others)	1250	32.16	130.93	75
*P. delicatulus*	(Others)	1400	32.30	131.43	76
*P. delicatulus*	(Others)	1320	32.28	131.43	77
*P. delicatulus*	(Others)	1250	31.94	130.85	78
*P. delicatulus*	(Others)	700	33.00	130.07	79
*P. delicatulus*	(Others)	900	32.98	130.09	80
*P. delicatulus*	(Others)	970	32.96	130.08	81
*P. delicatulus*	(Others)	1200	32.76	130.29	82
*P. kawadai*	No introgression	1400	36.44	138.64	83
*P. kawadai*	No introgression	1260	36.41	138.67	34
*P. kawadai*	No introgression	1400	36.20	138.64	35
*P. kawadai*	No introgression	1300	35.94	138.80	36
*P. kawadai*	No introgression	1120	35.91	138.82	37
*P. kawadai*	No introgression	1490	35.90	138.95	84
*P. kawadai*	No introgression	1400	35.87	139.09	85
*P. kawadai*	No introgression	1400	35.71	138.83	86
*P. kawadai*	No introgression	1550	35.56	138.75	87
*P. kawadai*	No introgression	1569	35.42	138.69	88
*P. kawadai*	No introgression	1550	35.38	138.53	48
*P. kawadai*	No introgression	1480	35.32	138.35	49
*P. kawadai*	No introgression	1400	35.64	138.35	89
*P. kawadai*	No introgression	1330	36.91	138.48	50
*P. kawadai*	No introgression	1350	36.11	138.65	90
*P. kawadai*	No introgression	1300	36.31	138.08	91
*P. kawadai*	No introgression	1550	35.39	137.99	52
*P. kawadai*	No introgression	1500	35.57	138.12	92
*P. kawadai*	No introgression	1600	35.57	138.08	93
*P. kawadai*	No introgression	1640	35.55	138.09	94
*P. kawadai*	No introgression	1600	35.44	137.96	95
*P. kawadai*	No introgression	1600	35.20	137.98	96
*P. kawadai*	No introgression	1600	35.24	137.96	97
*P. kawadai*	No introgression	1260	35.12	137.90	98
*P. kawadai*	Introgression	1460	35.52	138.97	99
*P. kawadai*	Introgression	1240	35.44	139.23	100
*P. kawadai*	Introgression	1210	35.48	139.17	41
*P. kawadai*	Introgression	1567	35.47	139.16	42
*P. kawadai*	Introgression	1600	35.48	139.10	101
*P. kawadai*	Introgression	1587	35.51	139.07	43
*P. kawadai*	Introgression	1673	35.49	139.14	102
*P. kawadai*	Introgression	1292	35.48	139.03	103
*P. kawadai*	Introgression	1400	35.51	139.05	44
*P. kawadai*	Introgression	1379	35.46	138.98	104
*P. kawadai*	Introgression	1320	35.40	138.92	105
*P. kawadai*	Introgression	1350	35.39	138.89	106
*P. kawadai*	Introgression	1420	35.23	139.02	107
*P. kawadai*	Introgression	1350	35.23	139.02	108
*P. kawadai*	Introgression	1299	34.86	139.02	109
*P. kawadai*	Introgression	1406	34.86	139.00	110
*P. kawadai*	Introgression	1200	34.85	138.96	111
*P. kawadai*	Introgression	1150	34.84	138.96	112
*P. kawadai*	Introgression	1013	34.84	138.89	113
*P. kawadai*	Introgression	1000	34.88	138.88	114
*P. akitaorum*		1420	34.36	136.09	115
*P. akitaorum*		1520	34.19	136.10	29
*P. akitaorum*		1450	34.27	135.94	116
*P. akitaorum*		1820	34.18	135.91	117
*P. sugitai*		1220	33.92	134.34	118
*P. sugitai*		1120	33.91	134.29	119
*P. sugitai*		1320	33.87	134.09	65
*P. sugitai*		1560	33.87	133.37	120
*P. sugitai*		1520	33.76	133.14	121

## APPENDIX 2 Samples of Platycerus used for morphological and genetic analyses

**Table:**

Species	Analysis Unit	Site No.	Number examined				Accession No. of DDBJ	
			Morphology		Genetic region			
			Male	Female	*COI*	ITS	*COI*	ITS
*P. delicatulus*	Allopatric	2	3	1	3	1	AB609374	LC651902
							LC651809	
							LC651810	
*P. delicatulus*	Allopatric	3	1	1	2	1	LC651811	LC651903
							LC651812	
*P. delicatulus*	Allopatric	9			3		AB609375	
							AB609376	
							AB609377	
*P. delicatulus*	Allopatric	10	3	3	2		AB426942	
							AB426943	
*P. delicatulus*	Allopatric	11	1					
*P. delicatulus*	Allopatric	12		1	1		AB426944	
*P. delicatulus*	Allopatric	13	16	8	4	1	AB609378	LC651904
							AB609379	
							AB609380	
							LC651813	
*P. delicatulus*	Allopatric	14	2	2				
*P. delicatulus*	Allopatric	15		5				
*P. delicatulus*	Allopatric	16	6	4	3	1	LC651814	LC651905
							LC651815	
							LC651816	
*P. delicatulus*	Allopatric	18	1	3	3	1	LC651817	LC651906
							LC651818	
							LC651819	
*P. delicatulus*	Allopatric	19	1	1				
*P. delicatulus*	Allopatric	21	2		2	1	LC651820	LC651907
							LC651821	
*P. delicatulus*	Allopatric	22			1		AB609381	
*P. delicatulus*	Allopatric	23	1		1		AB426951	
*P. delicatulus*	Allopatric	24	9	8	2		AB426952	
							AB426953	
*P. delicatulus*	Allopatric	29	1	2	3	2	AB609382	LC651908
							LC651822	LC651909
							LC651823	
*P. delicatulus*	Allopatric	31			1		AB609383	
*P. delicatulus*	Allopatric	33		1	1	1	LC651824	LC651910
*P. delicatulus*	Sympatric	34	5	7	3		LC651825	
							LC651826	
							LC651827	
*P. delicatulus*	Sympatric	35	1					
*P. delicatulus*	Sympatric	36	20	20	3		AB426945	
							AB426946	
							AB426947	
*P. delicatulus*	Sympatric	38		1				
*P. delicatulus*	Sympatric	39	5	5	3		LC651828	
							LC651829	
							LC651830	
*P. delicatulus*	Sympatric	40	3	4	3		LC651831	
							LC651832	
							LC651833	
*P. delicatulus*	Sympatric	44	20	9	1	2	LC651834	LC651911
								LC651912
*P. delicatulus*	Sympatric	46	1					
*P. delicatulus*	Sympatric	47	4	8	3		AB426948	
							AB426949	
							AB426950	
*P. delicatulus*	Sympatric	48	1	1	5		LC651835	
							LC651836	
							LC651837	
							LC651838	
							LC651839	
*P. delicatulus*	Sympatric	50		1	1		LC651840	
*P. delicatulus*	Sympatric	51	1	5	3	1	same as LC651840	LC651913
							LC651841	
							LC651842	
*P. delicatulus*	Sympatric	54	1	1	2	1	LC651843	LC651914
							LC651844	
*P. delicatulus*	(Others)	58			1	1	AB609384	LC651915
*P. delicatulus*	(Others)	59			1		LC651845	
*P. delicatulus*	(Others)	60			4	1	AB609385	LC651916
							AB609386	
							AB609387	
							AB609388	
*P. delicatulus*	(Others)	64			2		AB609389	
							AB609390	
*P. delicatulus*	(Others)	65			3	1	AB426954	LC651917
							AB609391	
							AB609392	
*P. delicatulus*	(Others)	67			1		LC651846	
*P. delicatulus*	(Others)	68			1		LC651847	
*P. delicatulus*	(Others)	69			1	1	LC651848	LC651918
*P. delicatulus*	(Others)	70			3	1	AB609393	LC651919
							AB609394	
							AB609395	
*P. delicatulus*	(Others)	71			2	1	LC651849	LC510902
							LC651850	
*P. delicatulus*	(Others)	72			3	1	AB609396	LC651920
							AB609397	
							AB609398	
*P. delicatulus*	(Others)	73			1		AB426955	
*P. delicatulus*	(Others)	74			1	1	LC651851	LC651921
*P. delicatulus*	(Others)	75			3		AB609401	
							AB609402	
							AB609403	
*P. delicatulus*	(Others)	76			2		AB609399	
							AB609400	
*P. delicatulus*	(Others)	77			2		LC651852	
							LC651853	
*P. delicatulus*	(Others)	78			4	2	AB609405	LC651922
							AB609406	LC651923
							AB609407	
							AB609408	
*P. delicatulus*	(Others)	79			2		AB426956	
							AB426957	
*P. delicatulus*	(Others)	80			1		AB426958	
*P. delicatulus*	(Others)	81			2	1	LC651854	LC510903
							LC651855	
*P. delicatulus*	(Others)	82			3	2	AB426959	LC651924
							AB426960	LC651925
							AB426961	
*P. kawadai*	No introgression	83	1	2	1	1	LC651856	LC510905
*P. kawadai*	No introgression	34	5	8	2	1	LC651857	LC651926
							LC651858	
*P. kawadai*	No introgression	35	1	1	1		LC651859	
*P. kawadai*	No introgression	36	13	5	3		AB426962	
							AB426963	
							AB426964	
*P. kawadai*	No introgression	37	2		2	1	LC651860	LC651927
							LC651861	
*P. kawadai*	No introgression	84	3	4				
*P. kawadai*	No introgression	85	6	3	4	1	LC651862	LC651928
							LC651863	
							LC651864	
							LC651865	
*P. kawadai*	No introgression	86	3	3	3	1	AB426965	LC651929
							AB426966	
							AB426967	
*P. kawadai*	No introgression	87	2	1	3	2	LC651866	LC651930
							LC651867	LC651931
							LC651868	
*P. kawadai*	No introgression	88	2	1	3	2	LC651869	LC651932
							LC651870	LC651933
							LC651871	
*P. kawadai*	No introgression	48			1		LC651872	
*P. kawadai*	No introgression	49	1		1	1	LC651873	LC651934
*P. kawadai*	No introgression	89	2	2	2		LC651874	
							LC651875	
*P. kawadai*	No introgression	50	1		1		LC651876	
*P. kawadai*	No introgression	90	2		2	1	LC651877	LC651935
							LC651878	
*P. kawadai*	No introgression	91		1	1		AB609408	
*P. kawadai*	No introgression	93	1	1	2		AB426968	
							AB426969	
*P. kawadai*	No introgression	94	10	17	1	1	LC651879	LC651936
*P. kawadai*	No introgression	97	2	3	2	1	LC651880	LC510906
							LC651881	
*P. kawadai*	No introgression	98	2	3	3	1	AB609409	LC651937
							AB609410	
							LC651882	
*P. kawadai*	Introgression	99	10	6	6	2	LC651883	LC651938
							LC651884	LC651939
							LC651885	
							LC651886	
							LC651887	
							LC651888	
*P. kawadai*	Introgression	100	4	7	2	1	LC651889	LC510904
							LC651890	
*P. kawadai*	Introgression	44	8	8	2	2	LC651891	LC651940
							LC651892	LC651941
*P. kawadai*	Introgression	105	12	9	2	1	LC651893	LC651942
							LC651894	
*P. kawadai*	Introgression	106	16	2	2	1	LC651895	LC651943
							LC651896	
*P. kawadai*	Introgression	107	16	9	2	2	LC651897	LC651944
							LC651898	LC651945
*P. kawadai*	Introgression	111	16	16	3	1	LC651899	LC651946
							LC651900	
							LC651901	
*P. kawadai*	Introgression	112	1					
*P. akitaorum*		115			1	1	AB609552	LC510919
*P. akitaorum*		29			1		AB427035	
*P. akitaorum*		116			1		AB427039	
*P. akitaorum*		117			1		AB609555	
*P. sugitai*		118			1		AB588791	
*P. sugitai*		119			1		AB588790	
*P. sugitai*		65			1	1	AB588793	LC510920
*P. sugitai*		120			1		AB588811	
*P. sugitai*		121			1		AB609559	
